# Sociodemographic, Psychosocial, and Health Behavior Risk Factors Associated with Sexual Risk Behaviors among Southeastern US College Students

**DOI:** 10.4236/ojpm.2014.46046

**Published:** 2014-06-01

**Authors:** Carla J. Berg, Kincaid Lowe, Erin Stratton, Sherell Brown Goodwin, Linda Grimsley, Jan Rodd, Catherine Williams, Cheri Mattox, Bruce Foster

**Affiliations:** 1 Department of Behavioral Sciences and Health Education, Emory University, Atlanta, USA; 2 Albany State University, Albany, USA; 3 Chattahoochee Technical College, Marietta, USA; 4 Central Georgia Technical College, Macon, USA

**Keywords:** Sexual Risk, Substance Use, College Students

## Abstract

**Objectives:**

We examined correlates of 1) being a virgin; 2) drug or alcohol use prior to the last intercourse; and 3) condom use during the last intercourse in a sample of college students.

**Methods:**

We recruited 24,055 students at six colleges in the Southeast to complete an online survey, yielding 4840 responses (20.1% response rate), with complete data from 4514.

**Results:**

Logistic regression indicated that correlates of virginity included being younger (p < 0.001), male (p = 0.01), being White or other ethnicity (p < 0.001), attending a four-vs. two-year school (p < 0.001), being single/never married (p < 0.001), lower sensation seeking (p < 0.001), more regular religious service attendance (p < 0.001), lower likelihood of smoking (p < 0.001) and marijuana use (p = 0.002), and less frequentdrinking (p < 0.001). Correlates of alcohol or drug use prior to most recent intercourse including being older (p = 0.03), being White (p < 0.01), attending a four-year college (p < 0.001), being homosexual (p = 0.041) or bisexual (p = 0.011), having more lifetime sexual partners (p = 0.005), lower satisfaction with life (p = 0.004), greater likelihood of smoking (p < 0.001) and marijuana use (p < 0.001), and more frequent drinking (p < 0.001). Correlates of condom use during the last sexual intercourse including being older (p = 0.003), being female (p < 0.001), being White (p < 0.001), attending a two-year school (p = 0.04), being single/never married (p = 0.005), being homosexual or bisexual (p = 0.04), and a more frequent drinking (p = 0.001).

**Conclusions:**

Four-year college attendees were more likely to be a virgin but, if sexually active, reported higher sexual risk behaviors. These nuances regarding sexual risk may provide targets for sexual health promotion programs and interventions.

## 1. Introduction

Young adults are likely to initiate risky health behaviors during college years. Sexual risk behaviors (e.g., number of partners, alcohol or drug use prior to intercourse, condom use) are particularly pervasive among college students (Lao, 2014; O’Malley, 2002). Most college students are or have been sexually active, with only 36.3% of students reporting being a virgin (American College Health Association, 2012) [1]. Furthermore, in the 12 months prior to the assessment, 75.8% of students having sex reported having multiple sexual partners (oral sex, vaginal or anal intercourse) (American College Health Association, 2012) [1]. With the majority being sexually active, the extent to which these young adults practice safe sex is critical to examine.

One safe sex practice involves consistent use of condoms or other contraceptives. The consistent and correct use of condoms is a potentially efficacious strategy to avert many of the sexually transmitted infections (STIs) commonly diagnosed in the United States (Centers for Disease Control and Prevention, 2010) [2]. Another important consideration is whether these young adults use substances that might influence their decision making and use of condoms. College students show risky substance use behaviors. About 15% of college students used cigarettes in the last 30 days with a third of those being daily users, a third (33.6%) of students reported having ever used marijuana with half of those reporting use in the last 30 days, and 74.5% of students reported having ever used alcohol with most students who used alcohol using 1–19 times in the last 30 days (57.2%) (American College Health Association, 2012) [1]. Overall, 24.9% and 21.9% of students reported doing something they regretted while they were drinking or forgot where they were or what they were doing while they were drinking respectively in the last 12 months (American College Health Association, 2012) [1]. This risky substance use behavior may also indicate concurrent risk behaviors, particularly sexual risk behavior. For example, 13.2% of students had unprotected sex while drinking alcohol with approximately the same distribution among between male and female respondents (American College Health Association, 2012) [1]. Prior research has suggested that adolescents who engage in one high risk behavior may also be likely to engage in other risky health behaviors. For example, young girls who smoke cigarettes are five times more likely than girls who do not smoke to experience early parenthood (Ellickson, Tucker, & Klein, 2001) [3]. Moreover, alcohol and tobacco are among the strongest predictors of an increased number of sexual partners among adolescent females (Valois, Oeltmann, Waller, & Hussey, 1999) [4]. Despite these findings, little research has documented psychosocial factors such as satisfaction with life, sensation seeking, or religious attendance in relation to sexual behavior, particularly among racially and ethnically diverse young adult college students.

Problem Behavior Theory (Jessor & Jessor, 1977) [5] suggests that multiple factors contribute to problem behaviors, defined as socially problematic, concerning, or undesirable behaviors usually eliciting some form of social or personal consequence (e.g., disapproval from others, incarceration, health compromise). The theoretical framework includes three major systems of explanatory variables: 1) the perceived-environment system, involving social controls, models, and support; 2) the personality system, involving values, expectations, beliefs, attitudes, and orientations toward self and society; and 3) the behavior system, encompassing both problem and conventional behaviors. Considering these explanatory systems, engaging in health-compromising behaviors such as sexual risk behaviors may be influenced by contextual factors that might be related to sociodemographic characteristics, psychosocial characteristics such as satisfaction with life, sensation seeking, and religious service attendance, as well as other risky behaviors such as substance use.

In light of this aforementioned research, the goal of the current study was to identify sociodemographic characteristics, psychosocial factors (*i.e.*, satisfaction with life, sensation seeking, religious service attendance), and substance use patterns related to three sexual behavior outcomes among a sample of racially/ethnically diverse young adult college students in the Southeastern US. Specifically, we examined the outcomes of lifetime abstinence from sexual intercourse, alcohol or drug use prior to most recent intercourse experience, and condom use during the most recent intercourse.

## 2. Methods

### 2.1. Procedure

In October 2010, students at six colleges in the Southeast were recruited to complete an online survey. A random sample of 5000 students at each school (with the exclusion of two schools who had enrollment less than 5000) were invited to complete the survey (total invited N = 24,055). Students received an e-mail containing a link to the consent form with the alternative of opting out. The consent form included information about the study (*i.e.*, cross-sectional survey about college student health), including the fact that their participation was strictly voluntary and that they could withdraw at any time without penalty. Students who consented to participate were directed to the online survey. To encourage participation, students received up to three e-mail invitations to participate. As an incentive for participation, all students who completed the survey were entered into a drawing for cash prizes of $1000 (one prize), $500 (two prizes), and $250 (four prizes) at each participating school.

Of students invited to participate, 4840 (20.1%) returned a completed survey. Consistent with our focus on young adults who may be initiating or escalating their smoking, the present study focused on students aged 18–30 years who also had complete sexual behavior data. Thus, the analyses were conducted on a final sample size of N = 4514. The Emory University Institutional Review Board approved this study, IRB# 00030631.

### 2.2. Measures

The online survey contained 230 questions assessing a variety of health topic areas, which took approximately 20–25 minutes to complete. For the current investigation, only questions related to sociodemographic characteristics, sexual behaviors, and the predictor factors of interest were included.

### 2.3. Sociodemographic Characteristics

We assessed students’ age, gender, ethnicity, highest parental educational attainment, and relationship status. Race/ethnicity was categorized as non-Hispanic White, Black, or other due to the small numbers of participants who reported other race/ethnicities. Relationship status was categorized as single/never married versus other. For ease of interpretation, these categorizations were chosen.

### 2.4. Sexual Behaviors

To assess sexual orientation, we asked, “What best describes your sexual orientation? Heterosexual; Homosexual; or Bisexual”. To assess sexual activity, we asked, “During your life, with how many people have you had sexual intercourse?” and “During the past 12 months, with how many people did you have sexual intercourse?” To assess two important indicators of sexual risk, we asked “Did you drink alcohol or use drugs before you had sexual intercourse the last time?” and “The last time you had sexual intercourse, did you or your partner use a condom?” with response options of “I have never had sexual intercourse; Yes; or No.”

### 2.5. Psychosocial Factors

To assess satisfaction with life, we administered the Satisfaction with Life Scale (Diener, Emmons, Larsen, & Griffin, 1985) [6], a five-item scale containing items such as “In most ways my life is close to my ideal” and “If I could live my life over, I would change almost nothing” on a scale of 1 = strongly disagree to 7 = strongly agree. The scale demonstrates appropriate validity and reliability (Cronbach’s alpha of 0.87). To assess sensation-seeking, we administered the Brief Sensation Seeking Scale-4 item (BSSS-4) (Stephenson, Hoyle, Palmgreen, & Slater, 2003) [7], which is an abbreviated version of the 8-item Brief Sensation Seeking Scale. The BSSS-4 includes the items from the BSSS after examining the psychometric properties of the BSSS (Hoyle, Stephenson, Palmgreen, Lorch, & Donohew, 2002) [8] and retaining one item from each of the four original subscales with the highest item-total correlation. Example items include “I would like to explore strange places” and “I like to do frightening things.” Psychometric analyses revealed appropriate internal consistency (Cron-bach’s alpha of 0.75), convergent validity, and test-retest reliability (Stephenson *et al*., 2003) [7]. We also asked participants to indicate the extent to which they agree with the statement “I frequently attend religious services” on a five-point scale of 0 = strongly disagree to 4 = strongly agree.

### 2.6. Substance Use

To assess other substance use, students were asked, “In the past 30 days, on how many days did you smoke a cigarette (even a puff)? Use marijuana (pot, weed, hashish, hash oil)? Drink alcohol? Drink more than 5 alcoholic drinks on one occasion?” These questions have been used to assess tobacco use in the American College Health Association (ACHA) surveys, National College Health Risk Behavior Survey (NCHRBS), and Youth Risk Behavior Survey (YRBS), and their reliability and validity have been documented by previous research (ACHA, 2008; CDC, 1997) [9] [10].

### 2.7. Data Analysis

Participant characteristics were summarized using descriptive statistics. Bivariate analyses were conducted comparing those who reported never having engaged in sex versus those who have. Then, among those who had previously engaged in sexual activity, we examined differences among those who reported using drugs or alcohol prior to the last intercourse versus not and among those who reported using a condom during the last intercourse versus not. We used chi-squared tests for categorical variables and t-tests for continuous variables. Binary logistic regression was used to determine factors associated with having previously been sexually active versus not among all participants. Then, among sexually active participants, we conducted binary logistic regression to determine factors associated with the two sexual behaviors of interest. Thus, we conducted three multivariate models. The predictors of interest (*i.e.*, sociodemographics, psychosocial factors, and substance use) were forced into each model. To account for number of previous sex partners in the regression models, we used lifetime number of sexual partners rather than past-year number of partners given the population of younger adults and relatively shorter sexual history for this group. To account for alcohol consumption in the regression models, we used number of days of alcohol use rather than binge drinking to account for frequency of consumption. SPSS 21.0 was used for all data analyses. Statistical significance was set at p = 0.05 for all tests.

## 3. Results

Table 1 presents participant characteristics and bivariate analyses examining differences 1) among those who reported never having engaged in sex versus those who have; 2) among sexually active participants who reported using drugs or alcohol prior to the last intercourse versus not; and 3) among sexually active participants who reported using a condom during the last intercourse versus not. Table 2 presents the logistic regression analyses for each of the sexual behaviors of interest.

### 3.1. Lifetime Sexual Activity

In terms of bivariate associations related to lifetime sexual activity (Table 1), significant findings were found across almost every dimension. Those who had never engaged in sexual activity in their lifetime were more likely to be younger (p < 0.001), male (p = 0.010), other ethnicity (p < 0.001), from a four-year school (p < 0.001), being single/never married (p < 0.001), and heterosexual (p = 0.021). They reported greater satisfaction with life (p = 0.022), lower sensation seeking (p = 0.001), and were more likely to report regular attendance at religious services (p < 0.001). In relation to substance use in the past 30 days, those who had never engaged in sexual activity were less likely to have used cigarettes (p < 0.001), used marijuana (p < 0.001), and binge drank (p < 0.001), and used alcohol less frequently (p = 0.001). Logistic regression (Table 2) indicated that significant correlates of virginity included being younger (p < 0.001), being male (p = 0.01), being White or other ethnicity (p < 0.001), attending a four-year school (p < 0.001), being single/never married (p < 0.001), lower sensation seeking (p < 0.001), more regular attendance at religious services (p < 0.001), lower likelihood of cigarette use (p < 0.001) and marijuana use (p = 0.002), and less frequently consuming alcohol (p < 0.001).

### 3.2. Alcohol or Drug Use Prior to Last Intercourse

Bivariate analyses (Table 1) indicated that those who had used alcohol or drugs prior to their last intercourse were more likely to be male (p < 0.001), White (p < 0.001), four-year college attendees (p < 0.001), single/never married (p < 0.001), and heterosexual (p < 0.001). They also reported higher satisfaction with life (p < 0.001), greater sensation seeking (p < 0.001), and more regular church attendance (p < 0.001). In terms of substance use in the past 30 days, those who had not used a condom during their most recent intercourse were more likely to use cigarettes (p < 0.001) and marginally more likely to use marijuana (p = 0.06). Binary logistic regression (Table 2) indicated that important correlates of alcohol or drug use prior to most recent intercourse included being older (p = 0.031), being White (p = 0.007 for Black, p = 0.002 for Other), being from a four-year college (p < 0.001), being homosexual or bisexual (p = 0.041 for homosexual; p = 0.011 for bisexual), having more lifetime sexual partners (p = 0.005), lower satisfaction with life (p = 0.004), greater likelihood of cigarette use (p < 0.001) and marijuana use (p < 0.001), and more frequently consuming alcohol (p < 0.001).

### 3.3. Condom Use in Last Intercourse

In terms of bivariate relationships (Table 1), those who used a condom during their last intercourse were younger (p < 0.001) and more likely to be female (p < 0.001), White (p < 0.001), two-year college attendees (p < 0.001), not single/never married (p < 0.001), and homosexual (p < 0.001). They also reported lower satisfaction with life (p < 0.001), lower sensation seeking (p < 0.001), and less regular church attendance (p < 0.001). In terms of substance use in the past 30 days, those who used a condom during their most recent intercourse were less likely to use cigarettes (p < 0.001), use marijuana (p < 0.001), and binge drink (p < 0.001), and less frequently consumed alcohol (p < 0.001). Regression analyses (Table 2) indicated that correlates of condom use during the last sexual intercourse included being older (p = 0.003), being female (p < 0.001), being White (p < 0.001), attending a two-year school (p = 0.040), not being single/never married (p = 0.005), being homosexual or bisexual (p = 0.040), and more frequently consuming alcohol (p = 0.001).

## 4. Discussion

This study documented sociodemographic characteristics, psychosocial factors, and substance use behaviors associated with ever having sex and, among those sexually active, substance use prior to last sexual intercourse and condom use at last sexual intercourse among a racially diverse college student population. Particularly important correlates of sexual risk behavior included race, type of school attended, sexual orientation, and substance use including past 30-day cigarette, marijuana, and alcohol use.

Sociodemographic characteristics were significantly associated with all three behavioral outcomes of interest and warrant elaboration. Those who were younger were more likely to be virgins, and if sexually active, were less likely to have used substances prior to intercourse. However, they were also less likely to have used condoms. Whites were less likely to be sexually active, but if active, were more likely to have used substances but also more likely to have used condoms. Interestingly, compared with two-year college students, four-year college attendees were more likely to be a virgin but, if sexually active, reported higher sexual risk behaviors. Recent literature shows that sexually active two year college students have higher sexual risk behaviors than their four-year college counterparts (Eisenberg, Lust, & Garcia, 2014) [11]. In this study, two-year college students were more likely to engage in only one of our two sexual risk behavior outcomes.

Relationship factors were also important. Being single was associated with being a virgin, while those in relationships were less likely to have used condoms. In terms of sexual orientation, being homosexual or bisexual was associated with substance use prior to sex, which is consistent with prior research (Mereish, O’Cleirigh, & Bradford, 2014) [12]. However, those reporting heterosexual orientation were also less likely to have used condoms.

We also found the anticipated associations with substance use and sexual risk behaviors. Specifically, substance use generally was associated with being sexually active, having used substances prior to intercourse, and lack of condom use, which is consistent with prior research (Simons, Maisto, & Wray, 2010) [13]. These findings add to the wealth of literature regarding the need to address substance use in order to reduce the likelihood of engaging in sexual risk behaviors (Centers for Disease Control and Prevention, 2010) [2].

Psychosocial factors only played a significant role in differentiating groups in the bivariate analysis and were less predictive of sexual risk behavior in the logistic regressions. Satisfaction with life, sensation seeking, and religious attendance were significantly different among students engaging in all three sexual behavior outcomes of interest. However, these psychosocial factors were significantly correlated only with virginity in the regressions. Virgins were more likely to attend religious services and report less sensation seeking behaviors compared to sexually active students. Psychosocial factors were not significantly correlated with alcohol use prior to last intercourse or condom use prior to last intercourse. This finding suggests that psychosocial factors may offer a protective effect for sexual initiation thereby mitigating or delaying some sexual risk behaviors among college students.

This study has implications for research and practice. In research, further studies are needed to understand the psychosocial and demographic factors that influence multi-risk behaviors among college students. Particularly important groups to study are students attending two versus four year colleges as well as sexually active versus non-sexually active students. In practice, health promotion program development needs to account for concurrent and multiple risk behaviors among collegiate populations. College is a time of emerging adulthood where new risk behaviors are often initiated and health behavior patterns are established (Wetherill, Neal, & Fromme, 2010) [14]. Initiation delay and risk reduction strategies are appropriate strategies to consider when developing health promotion programs or materials for collegiate populations.

### Limitations

Limitations to this study include limited generalizability due to recruitment at six colleges in the Southeast, with the participants being primarily female and White. This is important because minority smokers are more likely to be nondaily smokers. An additional limitation is the low response rate (20.1%), which may suggest response bias. However, previous research has found that the average email survey response rate is 24%, which is only slightly higher than the response rate for this survey (Sheehan, 2001). In addition, it is possible that some recruited students did not open the e-mail or had inactive accounts, which would influence the response rate. However, this cannot be assessed in the current study. Furthermore, previous research has indicated that, despite lower response rates, internet surveys yield similar data regarding health behaviors compared to mail and phone surveys (An, 2007). Despite these limitations, this study provides novel findings regarding sexual risk behavior among racially diverse college students.

## 5. Conclusion

In conclusion, our study shows that sociodemographic, psychosocial, and health behavior risk factors play a role in determining sexual risk behavior in students attending colleges in the Southeastern US. Consistent with existing literature, our data shows that college is a time when young adults may initiate or engage in risky health behaviors and interventions considering these factors are necessary to promote healthy behaviors and sexual risk reduction strategies. This analysis provides insight into specific individual factors like sexual orientation, school type, and substance use that are significantly associated with sexual risk behaviors. Given the variety of factors that influence sexual risk behavior, multi-risk behavior interventions may be necessary to target sexual risk behavior from multiple angles of influence.

## Figures and Tables

**Table 1:** Participant characteristics and bivariate analyses of sexual risk variables.

Variable	Total	Virgin			Used Alcohol or Drugs Prior to Last Intercourse			Used Condom during Last Intercourse		

	N (%) or M (SD)	No N (%) or M (SD)	Yes N (%) or M (SD)	p	No N (%) or M (SD)	Yes N (%) or M (SD)	p	No N (%) or M (SD)	Yes N (%) or M (SD)	p
*Sociodemographics*										
Age (SD)	23.54 (7.20)	24.13 (7.40)	20.40 (3.99)	<0.001	24.19 (7.50)	23.86 (6.91)	0.310	26.03 (5.80)	25.71 (8.45) <0.001	
Gender (%)				0.010			<0.001			<0.001
Male	1035 (28.7)	1035 (27.9)	259 (32.0)		796 (25.8)	239 (38.2)		576 (30.8)	459 (25.0)	
Female	3220 (71.3)	2670 (72.1)	550 (68.0)		2284 (74.2)	386 (61.8)		1296 (69.2)	1374 (75.0)	
Race (%)				<0.001			<0.001			<0.001
White	2056 (45.5)	1733 (46.8)	323 (39.9)		1378 (44.7)	355 (56.8)		752 (40.2)	981 (53.5)	
Black	1766 (39.1)	1463 (39.5)	303 (37.5)		1276 (41.4)	187 (29.9)		860 (45.9)	603 (32.9)	
Other	692 (15.3)	509 (13.7)	183(22.6)		426 (13.8)	83 (13.3)		260 (13.9)	249 (13.6)	
School Type (%)				<0.001			<0.001			<0.001
Four-year	2805 (62.1)	2171 (58.6)	634 (78.4)		1767 (57.4)	404 (64.6)		1251 (66.8)	920 (50.2)	
Two-year	1709 (37.9)	1534 (41.4)	175 (21.6)		1313 (42.6)	221 (35.4)		621 (33.2)	913 (49.8)	
Relationship Status (%)				<0.001			<0.001			<0.001
Single/Never Married	3337 (73.9)	2546 (68.7)	791 (97.8)		2070 (67.2)	476 (76.2)		1551 (82.9)	995 (54.3)	
Other	1177 (26.1)	1159 (31.3)	18 (2.2)		1010 (32.8)	149 (23.8)		321 (17.1)	838 (45.7)	
Sexual Orientation (%)				0.021			<0.001			0.020
Heterosexual	4218 (93.4)	3447 (93.0)	771 (95.3)		2899 (94.1)	548 (87.7)		1758 (93.9)	1689 (92.1)	
Homosexual	131 (2.9)	110 (3.0)	21 (2.6)		84 (2.7)	26 (4.2)		42 (2.2)	68 (3.7)	
Bisexual	165 (3.7)	148 (4.0)	17 (2.1)		97 (3.1)	51 (8.2)		72 (3.8)	76 (3.8)	
*Psychosocial Risk Factors*										
No. Partners in Lifetime (SD)	6.58 (13.25)	8.01 (14.23)	--	<0.001	7.10 (12.40)	12.52(20.48)	<0.001	6.76 (10.97)	9.29 (16.83)	0.471
No. Partners, Past 12 Months (SD)	3.27 (5.16)	1.84 (4.11)	--	<0.001	1.68 (3.93)	2.65 (4.83)	<0.001	2.04 (5.48)	1.64 (1.84)	0.131
Satisfaction with Life (SD)	22.20 (7.50)	22.08 (7.48)	22.81 (7.55)	0.022	22.28 (7.44)	21.03 (7.61)	<0.001	22.37 (7.36)	21.77 (7.60)	0.022
Sensation Seeking (SD)	3.32 (0.90)	3.34 (0.89)	3.22 (0.92)	0.001	3.30 (0.89)	3.58 (0.86)	<0.001	3.38 (0.87)	3.31 (0.91)	0.031
Religious Services Attendance (SD)	3.01 (1.48)	2.90 (1.45)	3.46 (1.48)	<0.001	2.98 (1.46)	2.52 (1.38)	<0.001	2.98 (1.42)	2.83 (1.49)	0.004
*Substance Use Risk Factors*										
Past 30-Day:										
Cigarette Use (%)				<0.001			<0.001			<0.001
No	3364 (76.3)	2635 (72.8)	729 (92.5)		2333 (77.4)	302 (49.8)		1382 (75.9)	1253 (69.6)	
Yes	1045 (23.7)	986 (27.2)	59 (7.5)		682 (22.6)	304 (50.2)		440 (24.1)	546 (30.4)	
Marijuana Use (%)				<0.001			<0.001			0.062
No	3771 (86.3)	3028 (84.4)	743 (94.5)		2671 (89.5)	357 (59.3)		1540 (85.4)	1488 (83.5)	
Yes	601 (13.7)	558 (15.6)	43 (5.5)		313 (10.5)	245 (40.7)		263 (14.6)	295 (16.5)	
High Risk Drinking (%)				<0.001			<0.001			0.132
No	3497 (77.5)	2766 (74.7)	731 (90.4)		2498 (81.1)	268 (42.9)		1413 (75.5)	1353 (73.8)	
Yes	1017 (22.5)	939 (25.3)	78 (9.6)		582 (18.9)	357 (57.1)		459 (24.5)	480 (26.2)	
No. Days Drinking Alcohol (SD)	3.27 (5.16)	3.71 (5.43)	1.29 (3.02)	0.001	2.85 (4.60)	7.96 (6.97)	<0.001	3.45 (5.08)	3.98 (5.76)	0.181

**Table 2:** Binary logistic regression indicating correlates of sexual behavior factors.

Variable	Virgin			Used Alcohol or Drugs Prior to Last Intercourse			Used Condom during Last Intercourse		

	OR	CI	p	OR	CI	p	OR	CI	p
*Sociodemographics*									
Age	0.89	0.86, 0.93	<.001	1.02	1.00, 1.04	0.031	1.02	1.01, 1.04	0.003
Gender									
Male	Ref	--	--	Ref	--	--	Ref	--	--
Female	0.68	0.56, 0.84	<0.001	1.07	0.85, 1.36	0.551	1.52	1.28, 1.81	<0.001
Race									
White	Ref	--	--	Ref	--	--	Ref	--	--
Black	0.49	0.40, 0.61	<0.001	0.65	0.50, 0.85	0.007	0.70	0.58, 0.83	<0.001
Other	1.17	0.91, 1.51	0.211	0.85	0.61, 1.19	0.002	0.93	0.74, 1.18	0.562
School Type									
Four-year	Ref	--	--	Ref	--	--	Ref	--	--
Two-year	0.63	0.50, 0.79	<0.001	0.76	0.58, 0.99	<0.001	1.20	1.00, 1.44	0.040
Relationship Status									
Single/Never Married	Ref	--	--	Ref	--	--	Ref	--	--
Other	0.07	0.04, 0.13	<0.001	0.56	0.42, 0.75	0.331	3.36	2.77, 4.07	0.005
Sexual Orientation									
Heterosexual	Ref	--	--	Ref	--	--	Ref	--	--
Homosexual	1.24	0.70, 2.19	0.471	1.68	1.00, 2.94	0.041	1.63	1.03, 2.58	0.040
Bisexual	0.87	0.47, 1.61	0.662	2.00	1.24, 3.23	0.011	0.87	0.58, 1.30	0.509
*Psychosocial Risk Factors*									
No. Partners in Lifetime	--	--	--	1.01	1.00, 1.02	0.005	1.01	1.00, 1.01	0.069
Satisfaction with Life	0.99	0.98, 1.00	0.051	0.99	0.97, 1.00	0.004	0.99	0.98, 1.01	0.264
Sensation Seeking	0.81	0.73, 0.90	<0.001	1.09	0.96, 1.24	0.192	0.99	0.91, 1.09	0.884
Religious Services Attendance	1.26	1.17, 1.34	<0.001	1.01	0.93, 1.10	0.792	0.97	0.92, 1.03	0.343
*Substance Use Risk Factors*									
Past 30-Day:									
Cigarette Use									
No	Ref	--	--	Ref	--	--	Ref	--	--
Yes	0.43	0.31, 0.59	<0.001	2.07	1.63, 2.62	<0.001	0.92	0.76, 1.11	0.403
Marijuana Use									
No	Ref	--	--	Ref	--	--	Ref	--	--
Yes	0.56	0.38, 0.81	0.002	3.57	2.77, 4.60	<0.001	1.02	1.00, 1.03	0.060
No. Days Drinking Alcohol	0.89	0.86, 0.92	<0.001	1.11	1.09, 1.13	<0.001	1.47	1.17, 1.84	0.001

## Abbreviations

OR: Odds Ratio
CI: 95% Confidence Interval
BSS-4: Brief Sensation Seeking Scale-4 Item
CDC: Centers for Disease Control and Prevention
ACHA: American College Health Association
NCHRBS: National College Health Risk Behavior Survey
YRBS: Youth Risk Behavior Survey
